# Five years of phenology observations from a mixed-grass prairie exposed to warming and elevated CO_2_

**DOI:** 10.1038/sdata.2016.88

**Published:** 2016-10-11

**Authors:** Melissa Reyes-Fox, Heidi Steltzer, Daniel R. LeCain, Gregory S. McMaster

**Affiliations:** 1 USDA-ARS, Soil Plant Nutrient Research Unit and Northern Plains Area, Fort Collins, Colorado 80526, USA; 2 Department of Biology, Fort Lewis College, Durango, Colorado 81301, USA; 3 USDA-ARS, Rangeland Resources Research Unit, Fort Collins, Colorado 80526, USA; 4 USDA-ARS, Agricultural Systems Research Unit and Northern Plains Area, Fort Collins, Colorado 80526, USA

**Keywords:** Grassland ecology, Climate change, Phenology

## Abstract

Atmospheric CO_2_ concentrations have been steadily increasing since the Industrial Era and contribute to concurrent increases in global temperatures. Many observational studies suggest climate warming alone contributes to a longer growing season. To determine the relative effect of warming on plant phenology, we investigated the individual and joint effects of warming and CO_2_ enrichment on a mixed-grass prairie plant community by following the development of six common grassland species and recording four major life history events. Our data support that, in a semi-arid system, while warming advances leaf emergence and flower production, it also expedites seed maturation and senescence at the species level. However, the additive effect can be an overall lengthening of the growing and reproductive seasons since CO_2_ enrichment, particularly when combined with warming, contributed to a longer growing season by delaying plant maturation and senescence. Fostering synthesis across multiple phenology datasets and identifying key factors affecting plant phenology will be vital for understanding regional plant community responses to climate change.

## Background & Summary

Climatic change factors, including increasing temperatures and rising CO_2_ levels, have been shown to affect plant phenology^1–3^. We investigated the effects of warming and CO_2_ enrichment on the timing and duration of four key developmental phases (phenophases) and found, in a mixed-grass prairie, the indirect effects of warming and CO_2_ enrichment on soil water availability play a central role in governing whether plants expedite or prolong development. If sufficient water resources exist, and temperatures remain above minimum thresholds, species will persist later into the season. If not, they often mature and senesce more quickly. Independent shifts in the magnitude and direction of life histories are species-specific, which in our system means the duration of the growing and reproductive seasons can be attributed to the response of one or two species per year (not necessarily the same species), and not a community-wide tendency to either extend or shorten growth and reproduction. Shifts in species’ life histories can influence plant community composition by disrupting timing sensitive relationships between plants and their associated pollinators and herbivores, and by decreasing the level of species’ complementarity. Variations in the timing of resource use can have detrimental effects on plant communities by facilitating invasive species colonization and limiting nutrient uptake/cycling via reductions in terrestrial biomass production^4,5^.

Warming has the potential to extend the growing and reproductive seasons by promoting earlier leaf and flower emergence^5–7^. However, those species showing significant advancements in leaf emergence and flower production in response to warming also matured and senesced earlier. We provide evidence that warming causes earlier leaf emergence but the magnitude of this advancement was not as great as the delay in senescence that resulted from exposure to combined warming and CO_2_ enrichment^8^ (see Reyes-Fox and Steltzer *et al.* 2014 for the full *Nature* article). Mean growing season duration was lengthened by 6.2 (+/−8.0) days under warming alone versus 14.2 (+/−7.0) days under combined warming and CO_2_ enrichment, indicating warming may not be the only, or even primary, phenological cue in prolonging the growing season^9,10^. CO_2_ enrichment has been shown to play an important role in limiting stomatal aperture and thus minimizing water loss via transpiration. Experimental research shows evidence that the water savings resulting from CO_2_ enrichment counteracts the desiccating effect of moderate warming^8,11^. Where direct effects of CO_2_ enrichment on plant phenology are concerned, the conclusions vary greatly. A number of studies focus on measures of reproductive investment such as floral abundance and mass, seed viability, etc. (refs 12–14), but not on the effect on the timing of leaf production or senescence. Research suggests elevated CO_2_ affects the timing of plant reproduction variably, expediting flowering in some species and delaying them in others^15^ and it has been shown to stimulate growth directly by enhancing photosynthetic rates and thus biomass production^16–18^. Many plants must reach a minimum size before a secondary cue (e.g., daylength) will trigger flowering. CO_2_ can directly affect flowering time via this mechanism but the magnitude and direction of this effect is difficult to predict^19,20^.

The authors present this phenology dataset and the accompanying microclimate data, which have reuse value in the fields of plant ecology and climate change. The raw data have implications for developing models that use environmental cues to predict the timing of phenology events for important developmental stages to test whether common patterns emerge from multiple datasets across different sites. There is also potential to use the data to conduct sensitivity analyses to determine thresholds for volumetric soil water content (VSWC) in different systems or to create thermal time estimates of species growing degree-day (GDD) responses for different developmental stages. Synthesis analyses across multiple phenology datasets will be useful in identifying key factors affecting plant phenology (e.g., late season VSWC in our case) and illuminating areas where uncertainty exists. The data will also be relevant to land managers interested in the timing of annual life cycle events to determine best management practices (prescribed burning, for example) and ranchers who rely on predictions of aboveground biomass production to determine stocking rates. Phenology data can also be used by those afflicted by seasonal allergies, or who are interested mutualisms, designations of migratory corridors, or any other area where the timing of life history events is important.

## Methods

### Site description

The Prairie Heating and Carbon Dioxide Enrichment (PHACE) experiment, initiated in 2006, is located west of Cheyenne, WY, USA at the USDA-ARS High Plains Grasslands Research Station in the U.S. Great Plains (41° 11’ N, 104^o^ 54’ W, elevation 1,930 m). This is a Northern mixed-grass prairie ecosystem, with a plant community comprised of 55% cool-season grasses, 25% warm-season grasses, and 20% sedges, forbs, and small shrubs. Total annual precipitation averages 38.5 cm and mean daily air temperatures range from −2.5 ^o^C in January to 17.5 ^o^C in July. The average wind speed is 6 m s^−1^ with gusts up to 35 m s^−1^. The site is comprised of two distinct soil types: an Ascalon Variant Loam (fine-loamy, mixed mesic) at the north end of the field and an Altvan Loam (fine-loamy over sandy, mixed mesic) at the south end. The 2.4 hectare site has a history of moderate grazing from 1928 until 2005, when fences were installed to prevent cattle from entering.

### Experimental design

**Figure 1:**
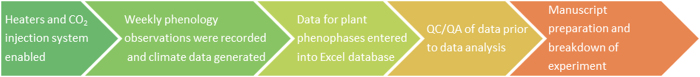
Experimental workflow diagram of PHACE phenology study.

**Figure 2:**
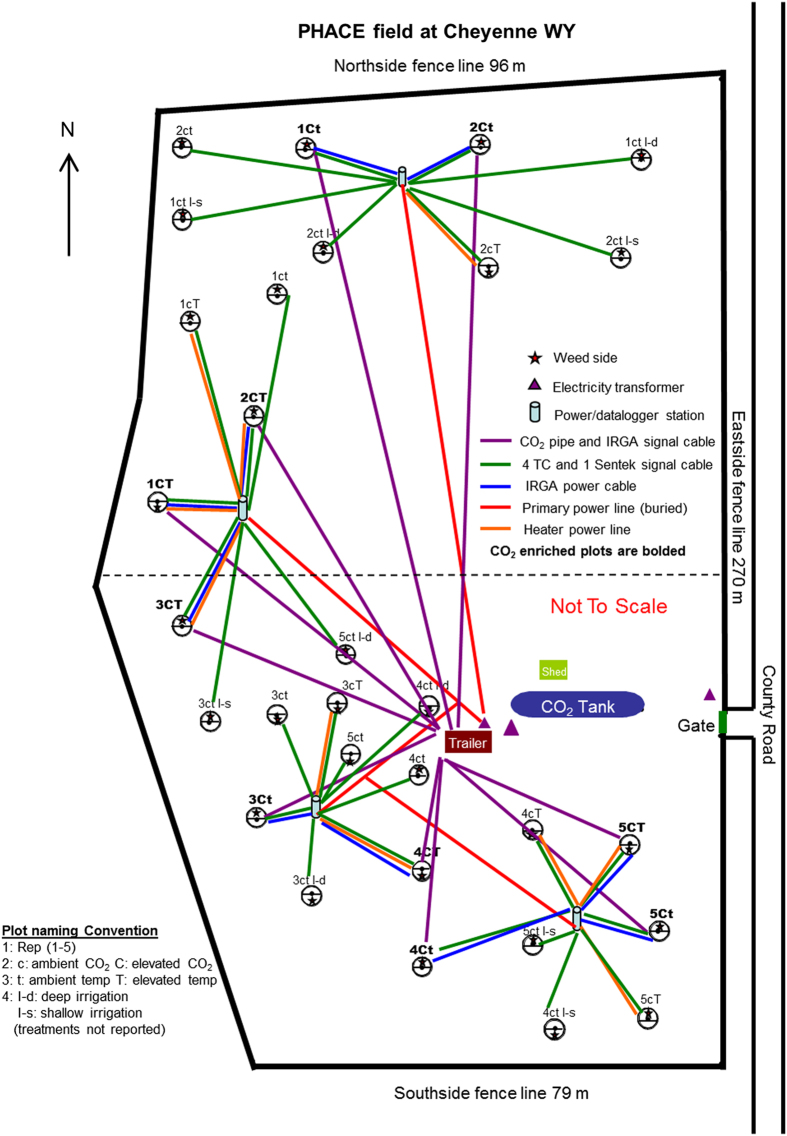
Layout of PHACE experimental treatment plots.

The experiment includes two levels of temperature (ambient and warmed, 1.5/3.0 °C warmer day/night, treatments t and T, respectively) and two levels of atmospheric CO_2_ concentrations (ambient 385 ppmv and elevated 600 ppmv CO_2_, treatments c and C, respectively) in a factorial combination with five replicate plots per treatment (ct, cT, Ct, and CT) for a total of 20 plots. Differential day/night time temperatures were implemented because minimum temperatures are predicted to increase at a faster rate than maximum temperatures^21^. Treatment combinations of ambient and elevated temperatures and CO_2_ concentrations are modelled after current climate change projections based on moderate, continued increases in CO_2_ for the end of this century^22^. Warming and elevated CO_2_ treatments were randomly assigned to the 3.3 m diameter circular plots (Fig. 1 depicts an outline of the approach used in this experiment). T-FACE technology for increasing temperature was implemented in spring of 2007 and warmed plots year round for the duration of the experiment^23^. Dummy heaters were installed in non-heated plots to eliminate response differences that may result from shading or other influences caused by the heating apparatuses. Free air CO_2_ enrichment (FACE) technology was used for enriching CO_2_ and began in 2006 (ref. 24). The CO_2_ fumigation system ran continuously during the daylight hours of the growing season (typically from about 1 April to 15 November), but was turned off after the plants ceased growing each year. Figure 2 depicts the experimental layout of the entire PHACE experiment; including the 10 irrigated plots (where phenology data was collected but is not reported here).

### Observations

The timing of four life cycle events that determine the start and end of species’ active and reproductive periods (leaf emergence, flower production, seed maturation, and canopy senescence) was observed weekly for six common species. The most abundant species in each growth form were chosen, including the one sub-shrub *Artemisia frigida, L.* (ARFR); a warm-season grass *Bouteloua gracilis, Lag. ex Griffiths* (BOGR); three cool-season grasses *Hesperostipa comata*, (*Elias) Barkworth* (HECO), *Koeleria macrantha*, *(Ledeb.) Schult* (KOMA), and *Pascopyrum smithii, (Rydb.) Á. Löve* (PASM); and a widespread forb (*Sphaeralcea coccinea, (Nutt.) Rydb.* (SPCO). Leaf emergence was characterized by the first new, green leaf to appear on a shoot. Flower production was reached when the first open flower (ARFR and SPCO) or inflorescence emerged from leaf sheath (BOGR, HECO, PASM, KOMA). Maturation was characterized as spikes subtending below the inflorescence which detach easily for BOGR; no green colour on seed heads for PASM and KOMA; wilted/dead flowers for ARFR and SPCO; seeds are unsheathed in HECO and are easily separated from the peduncle/stalk of the inflorescence. Senescence was reached when less than 10% green colour in the entire plant remained and plant growth ceased. Some species (i.e. ARFR) can overwinter and therefore will never be entirely brown.

To conduct phenology observations, a sample grid was placed over the centre area of the plot, and an access platform was used so the observer could hover over each of the 20 plots. The 3.3 m diameter circular plots were subdivided into two halves: one supporting the native prairie vegetation and the other dedicated to a separate invasive plant study. The far end of the wooden platform was set upon a metal flange located between the northern mixed-grass prairie study area and the weed observation area. The other end of the platform fell outside the CO_2_ injection tubing which circled the perimeter of the plot, thereby avoiding damage to the plots. Positioning the platform above the plots ensured the observer could view the plants up close. During years 2007–2009, the base of each individual was marked with a headpin and those same marked individuals were re-visited every week and their developmental phase was recorded for the duration of the growing season. Non-grass species (i.e., ARFR and SPCO) were not marked because they were easily identified. To ensure spatial variability we tried to identify one individual per species in each of the 24 quadrats of the sample grid. New individuals were numbered sequentially and added to the database. Typically at least one individual from BOGR, PASM, and HECO were found in each of the quadrats. Sample sizes for KOMA, ARFR and SPCO varied greatly between plots with an average of five individuals per plot per species. From 2007–2009, if a particular individual plant did not re-emerge after over-wintering it was replaced with another individual plant selected from nearby. If an alternate plant could not be located, the individual was removed from the list. Using maps for each plot, ID numbers for new individuals were assigned and arranged on the maps and given a distinguishing characteristic (i.e., bold or italics) depending on their current phenophases. This protocol was also followed if previously marked individuals were missing or senesced. In 2010 and 2011, the methods changed slightly and the plot maps were no longer used to track phenostages. Instead of tracking phenostages of individual plants for each target species, we used a ‘threshold’ to identify when a species had reached a particular phenostage. For instance, we surveyed the sample area and when we could see 10 BOGR individuals that had reached leaf emergence, we recorded it. Less common species like ARFR had a threshold of 5. In 2010 and 2011 the same phenostage criteria were used except heading in grasses was also defined, where possible. Over the course of the study, observers differentiated between senesced and non-existent individuals.

## Data Records

This was an experimental study where observations were conducted from 2007 through 2011, and where no samples were collected from within the plots nor were laboratory analysis conducted. Data outputs for plant individuals, climate and VSWC were generated and the reader is referred to corresponding data files (Table 1) where primary data and metadata are archived. All data were entered into an Excel spreadsheet and Proc Mixed (SAS version 9.2, 2008, SAS Institute, Cary, NC) was used to analyse the data (see Reyes-Fox and Steltzer *et al.* 2014 for full disclosure of statistical analyses). Plot markers and infrastructure were removed at the conclusion of the experiment.

### Phenology

From 2007–2011 all plots were clearly delimited and weekly phenology observations were recorded within each of the 20 plots from mid-March to early November. We used observational data from this time period to determine changes in growing and reproductive season length. Data are presented annually and across years for the duration of species’ active and reproductive periods (Data Citation 1, PHACEphenology_database_final_forSD.xlsx). The start and end of the growing season were characterized by the mean across replicate plots for leaf emergence by the first species to leaf and for canopy senescence by the last species, respectively. Similarly, the start and end of the reproductive season were characterized by the date when the first species flowered and when the last species reached seed maturation, respectively. Table 2 (available online only), reports replicate means in DOY for the 4 observed phenostages by year and by species. Standard errors and sample sizes are also provided.

### Climate and VSWC

Mean daily temperature and precipitation were calculated based on half hourly data from a meteorological station (HOBO, Onset, Inc., MA) at the field site (Data Citation 1, PHACEclimate_database_final_forSD.xlsx). In some cases (namely in 2011) missing data was replaced by data from a proximate met station that recorded hourly data instead of half hourly data. The substitute met station was the High Plains Grassland Research Station located approximately 1 km south of the PHACE pasture. It was a Campbell Scientific system and therefore had different sensors and datalogger programing. In the winter of 2009–2011, precipitation amounts were adjusted to account for moisture received as snow versus rain but this correction was done to daily averages, not the half hourly data. In 2009–2011, daily precipitation totals were adjusted to account for differences in snowfall versus rainfall using a snowfall adapter at the HPGRS. Prior to 2009, an accurate means of measuring snow moisture equivalents was not available. In our case, site-specific climate data was used to depict seasonal variation in precipitation and air temperature among years. When placed in a historical context of the last century, below average precipitation fell in 2007, 2008, and 2010 with above average precipitation falling in 2009 and 2011. 2010 was the warmest and driest of the five years and 2011 was the wettest year, however temperatures in 2009 were the coolest.

In each plot, the VSWC was measured hourly at 10, 20, 40, 60 and 80 cm depths (EnviroSMART probe: Sentek Sensor Technologies, Stepney, Australia). However, we present only the fall soil water measurements here, since significant treatments differences at this period of the growth season were determined to affect plant phenology^8^. Daily means were calculated for soil water content at the primary rooting depth (5–25 cm) by averaging the values for the sensors at 10 and 20 cm depth (Data Citation 1, PHACEswc_database_final_forSD.xlsx). Interannual variation in microclimate interacted with species phenology to produce dramatic differences in VSWC, particularly at the end of the season, among years.

## Technical Validation

### Quality assurance and control

**Figure 3:**
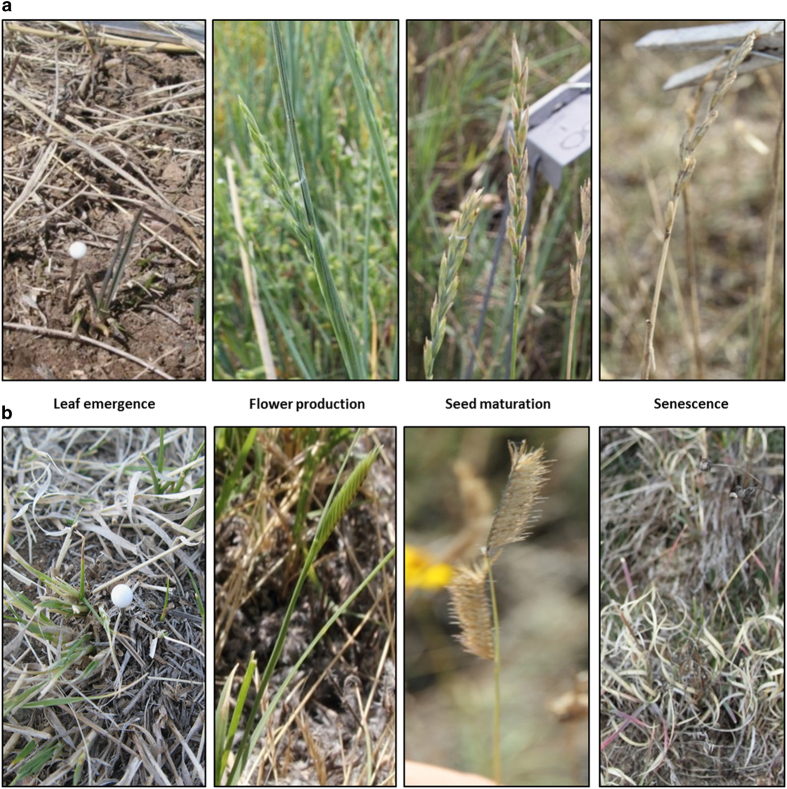
Pictorial index of plant phenophases for (**a**) C-3 grass (*Pascopyrum smithii*) and (**b**) C-4 grass (*Bouteloua gracilis*). Photo credits Daniel LeCain and Julie Kray.

From 2007–2009 individual plants were marked, species were color-coded and these marked individuals were re-visited to avoid misidentification. This was particularly helpful when the plants were first emerging and looked similar. If the pins were non-detectable, the observer referred to the field maps, where the location of each individual plant was recorded. If the specific location of a particular plant was not found, an alternate, nearby individual was marked. We minimized misidentification by executing thorough training in species identification. This included a combination of *in-situ* plant identification training by data observers and use of photographic plant guides for visuals of phenological stages for each species (Fig. 3 gives an example for two species). To ensure quality of data collection (i.e., observations), we also harvested sample specimens of each species from outside of the plots, archiving them in their vegetative and reproductive states to reference as needed. Samples were not collected for physical or chemical analysis of any kind. In 2010 and 2011, instead of following the same individual plants, the timing of an event was achieved when a minimum number of individuals for each species within a plot had completed a life history event, representing the median value. Despite this difference in approaches, both methods represented a central tendency to quantify event timing across multiple individuals per plot. We performed a direct one to one comparison of treatment means presented in Table 2 (available online only) for Method 1 (2007–2009) versus Method 2 (2010–2011) at each growth stage averaged across species for each method. A linear regression analysis plotting one method on the x-axis and the other on the y-axis against produced a R^2^ value of .98.

Data were recorded and updated weekly, and were checked for inconsistencies. We also made certain data fields were accurately labelled. To ensure treatments (i.e., heaters and CO_2_ injection system) were operating properly, data loggers were installed at PHACE and were routinely monitored^11,25^. Species-level phenological trends varied from year to year but the likelihood that this variation was due to observer error is low since a standard reporting protocol was implemented and data were collected over the course of the five year study by the same 3 persons. Also, treatment level effects were not as variable as the within-plot species-level variation.

T-FACE technology for increasing temperature began on April 10th 2007, after leaf emergence by the cool-season grasses and shrubs. Since warming began after the first species leafed, leaf emergence data were omitted for all species in 2007 and growing season length was not calculated. Similarly, some species did not flower in all years. In 2009, flower production measurements were not collected from the control (ct) and CO_2_ enriched plots (Ct) for BOGR, and seed maturation was not recorded for ARFR or SPCO in any of the treatments. Therefore reproductive season length could not be determined for this particular year.

## Additional Information

**How to cite**: Reyes-Fox, M. *et al.* Five years of phenology observations from a mixed-grass prairie exposed to warming and elevated CO_2_. *Sci. Data* 3:160088 doi: 10.1038/sdata.2016.88 (2016).

## Supplementary Material



## Figures and Tables

**Table 1:** Location and description of primary databases.

**Data Record**	**Source**	**Sample**	**Number of data values**	**Temporal range**	**Protocol 1**	**Protocol 2**	**Data file name**	**Repository**	**Size**
1	Reyes-Fox, M., Steltzer, H., LeCain, D. R., McMaster, G. S. *Dryad Digital Repository* http://dx.doi.org/10.5061/dryad.267d2 (2016)	Phenology	117635	2007–2011	Plot-level phenology observations	Method to identify treatment differences	PHACEphenology_database_final_forSD.xlsx	*Dryad*	587.9 Kb
2	Reyes-Fox, M., Steltzer, H., LeCain, D. R., McMaster, G. S. *Dryad Digital Repository* http://dx.doi.org/10.5061/dryad.267d2 (2016)	Climate	359630	2007–2011	Site-level climate data	Method to test interannual variability	PHACEclimate_database_final_forSD.xlsx	*Dryad*	2.299 Mb
3	Reyes-Fox, M., Steltzer, H., LeCain, D. R., McMaster, G. S. *Dryad Digital Repository* http://dx.doi.org/10.5061/dryad.267d2 (2016)	Volumetric Soil Water Content	7307	2007–2011	Plot-level soil water measurements	Method to identify treatment differences	PHACEswc_database_final_forSD.xlsx	*Dryad*	76.64 Kb

**Table 2:** Mean timing of life cycle events (in DOY) for all species in all treatments in all years

**Species**	**Year**	**Treatment**	**n**	**Leaf emergence**	**Leaf emergence SE**	**Flower production**	**Flower production SE**	**Seed maturation**	**Seed maturation SE**	**Canopy senescence**	**Canopy senescence SE**
Arfr	2007	ct	5	—	—	238.50	1.80	257.4*	3.50	336.7*	2.20
Arfr	2007	cT	5	—	—	243.50	0.70	255.6*	2.40	331.60	7.80
Arfr	2007	Ct	4	—	—	238.00	2.00	250.8*	0.80	326.9*	8.30
Arfr	2007	CT	4	—	—	243.30	1.50	252.6*	1.30	336.8*	2.80
Arfr	2008	ct	5	87	3.10	261.30	3.90	274.9*	1.50	312.20	6.90
Arfr	2008	cT	5	90.7	6.40	266.20	8.00	276.7*	6.20	306.10	12.00
Arfr	2008	Ct	4	88.4	3.60	261.00	1.00	274.3*	0.70	325.7*	4.30
Arfr	2008	CT	4	81.1	2.80	260.00		270.5*		291.20	10.70
Arfr	2009	ct	5	82.1	3.90	260.70	2.70	—		311.00	5.30
Arfr	2009	cT	5	77.5*	1.00	259.40	2.50	—		310.5*	9.70
Arfr	2009	Ct	5	79.3*	2.20	264.60	6.00	—		333.3*	2.00
Arfr	2009	CT	5	83.3	1.60	261.30	3.60	—		339.9*	1.40
Arfr	2010	ct	5	95.2	2.00	236.20	2.00	259.6*	2.20	295.6*	2.20
Arfr	2010	cT	5	73.4	4.70	235.00	1.20	261.0*	3.30	296.0*	5.20
Arfr	2010	Ct	5	93.6	1.40	238.50	1.50	262.3*	3.90	289.50	6.60
Arfr	2010	CT	5	75.3	5.50	235.30	1.70	259.0*	3.00	298.3*	3.90
Arfr	2011	ct	5	93.8	2.90	227.20	1.10	278.80	2.80	295.50	4.20
Arfr	2011	cT	5	88.4	3.30	228.00	1.00	276.0*	0.00	304.0*	0.00
Arfr	2011	Ct	5	93.5	2.90	235.00	2.10	279.5*	3.50	297.0*	2.90
Arfr	2011	CT	5	88	2.60	228.80	1.80	278.0*	2.00	300.5*	3.50
Bogr	2007	ct	5	—		229.3	1.5	254.9	2.5	291.8	3
Bogr	2007	cT	5	—		227.3	0.9	252	1.8	299.7	2
Bogr	2007	Ct	5	—		228.6	1.1	249.6	0.9	304.1	1
Bogr	2007	CT	5	—		223.4	1.6	249.1	2.7	300.5	1.8
Bogr	2008	ct	5	116.1	1.2	241.7	1.8	268.3	0.7	303	0.5
Bogr	2008	cT	5	107.7	0.5	242.9	3.1	271.2	0.7	298.9	2.1
Bogr	2008	Ct	5	115.9	0.8	248	5.2	272.1	4.1	306.7	0.5
Bogr	2008	CT	5	107.7	1.7	240.2	2.4	263.8	2	309.9	1.6
Bogr	2009	ct	5	106.2	0.9	—		—		272.9	5.1
Bogr	2009	cT	5	105.4	0.7	210.7		223.0*		261.9	4.3
Bogr	2009	Ct	5	106.9	1.6	—		—		280.8	5.2
Bogr	2009	CT	5	106.2	1	204		—		269.3	6
Bogr	2010	ct	5	110.6	1.6	197.6	2.2	222.6	0.6	269.2	1.7
Bogr	2010	cT	5	86.4	6.7	192.8	3.6	215.8	0.7	260.2	5
Bogr	2010	Ct	5	112.2	2	202.4	2.5	221.6	2.2	271.6	2.9
Bogr	2010	CT	5	89.4	6.2	194	4.1	218.8	2	274.8	4.8
Bogr	2011	ct	5	115	0	220.6	2	239	3.5	292.8	1.7
Bogr	2011	cT	5	101	0	222.5	0.9	242.2	2.3	276.6	9.1
Bogr	2011	Ct	5	113.6	1.4	216.8	4.8	233.4	2.9	288.6	3.4
Bogr	2011	CT	5	101	0	212.8	6.2	234.2	4	298.4	1.4
Heco	2007	ct	5	—		184.7	0.7	205.2	3.6	318.8	0.2
Heco	2007	cT	4	—		179.4	2	206.6	3.4	333.4*	2
Heco	2007	Ct	4	—		184.2	3.6	202.6	3.8	325.8	1.1
Heco	2007	CT	5	—		179.4	2.1	197.3	1.4	333.2	2.2
Heco	2008	ct	5	89.3	0.8	180.8	5.1	203.1	4.1	320.7	3.3
Heco	2008	cT	4	82.5	1.1	174.5	1.9	193	1.8	325.8	3.4
Heco	2008	Ct	5	86.7*	0.9	181.9	4.4	205.4	4.8	317.4	7.2
Heco	2008	CT	5	85.2	0.6	175.3	1.9	194.2	1.3	331.1	0.8
Heco	2009	ct	5	81.5	1.7	188.2	3.2	204	0	267.5	14.4
Heco	2009	cT	4	78	1.6	189.5	3.8	204.5	0.4	297.5	7.8
Heco	2009	Ct	4	85.7	1.3	194.6	1.9	204.3	0.3	310.3	4.5
Heco	2009	CT	5	79.1	0.8	184.6	2.6	204.5	0.3	307.9	7.3
Heco	2010	ct	5	92.5	4	192.5	1.7	203.3	0.8	293.5	4.3
Heco	2010	cT	5	72.2	3	172.2	5.8	198.6	0.6	289.2	5.5
Heco	2010	Ct	5	95	0	185.8	3.6	201	1.3	292.0*	3.7
Heco	2010	CT	5	68.2	2.2	170	3	198	0	290.2	3
Heco	2011	ct	5	97.4	2.4	186.2	3.9	198.2	1.6	280.2	4.2
Heco	2011	cT	5	83.2	0.8	170.8	3.5	188.5	1.7	278	2
Heco	2011	Ct	5	91.6	2.9	183.2	4.3	197.4	1.6	279.2	2
Heco	2011	CT	5	81.6	1	168	1.8	187.2	0.7	279.2	2
Koma	2007	ct	4	—		158.0*	7.8	185.7	4.1	329.5	8.3
Koma	2007	cT	4	—		145.2*	1.7	184.9	1.7	322.8	10.6
Koma	2007	Ct	4	—		156.3*	5	190	5	307	31
Koma	2007	CT	2	—		167.2		185.8		309.8	0.8
Koma	2008	ct	5	85.9*	1.3	158.8*	2.5	178.7	1.4	322.6*	5.9
Koma	2008	cT	3	76.3*	3.3	153.8*	3.8	173.5	3.5	291.2	8
Koma	2008	Ct	3	93.4	4.7	158.3*	4.3	175.6	1.4	324.6	11
Koma	2008	CT	5	80.9*	1.3	156.7*	2.5	173.7	1.6	332.0*	5
Koma	2009	ct	4	78.4*	1.7	155.7*	4.4	186.4	1.7	317.4*	8.7
Koma	2009	cT	4	87.5	6.8	145.6*	0.7	187.9	1.1	292.2	2.9
Koma	2009	Ct	5	82.3	2.7	149.0*	1.6	187.5	5.5	321.5	14.6
Koma	2009	CT	5	93.4	4.4	144.9*	2.1	197.2	5.2	320.2	13.4
Koma	2010	ct	5	73.0*	3.4	149.6*	1	206	1.2	275	1.7
Koma	2010	cT	5	67.0*	1	145.0*	0	209	0	268.4	4.3
Koma	2010	Ct	5	70.4*	2.7	151.4*	2.1	208	1	274.4	1.5
Koma	2010	CT	5	68.2	2.2	147.8*	1.7	209	0	273	2.4
Koma	2011	ct	5	80.8*	1.5	156.0*	1.4	190	1.3	287.2	6.9
Koma	2011	cT	5	76.8*	0.8	146.6*	2.7	185.2	0.8	287.2	6.9
Koma	2011	Ct	5	81.6*	1.6	156.6*	1.3	190.8	2.6	291.4	6.4
Koma	2011	CT	5	76.8*	0.8	144.2*	1.7	185.8	1.1	278.7	2.7
Pasm	2007	ct	5	—		192.5	7.5	252	9	295.3	3.1
Pasm	2007	cT	5	—		193		228		288.7	8.2
Pasm	2007	Ct	5	—		190		233.3		291.5	4.6
Pasm	2007	CT	5	—		168		222		302.9	6.2
Pasm	2008	ct	5	98.2	3.7	194.5	4.5	212	0	304.7	2.5
Pasm	2008	cT	5	86.7	2.7	184		205		282.6	8.9
Pasm	2008	Ct	5	96.5	2.5	199		221		297.9	5.1
Pasm	2008	CT	5	94	5.9	188		205		296.3	3.9
Pasm	2009	ct	5	85.6	1.6	196		211		270.7	5
Pasm	2009	cT	5	86.4	4	206.8	5.6	221.3	1.7	270.3	3.4
Pasm	2009	Ct	5	88.8	3.3	204		217.0*		273.6	7.4
Pasm	2009	CT	5	82.8	1.5	196	0	220.0*	3	276.8	6.3
Pasm	2010	ct	5	99	0	188.8	3.3	233.7	1.7	261.6	3.9
Pasm	2010	cT	5	77	5.4	193.3	10.7	227.3	5.8	254.6	5.2
Pasm	2010	Ct	5	101.6	1.3	—		—		259	6.1
Pasm	2010	CT	5	83		193		237		256.6	2.4
Pasm	2011	ct	5	90.4		194.5	1.5	246	2	280.2	4.2
Pasm	2011	cT	5	80.8		174	1	250.3	4.7	274	8.8
Pasm	2011	Ct	5	97.6		175		217		287.4	3.5
Pasm	2011	CT	5	81.6		183	6	230.5	13.5	277.4	5.1
Spco	2007	ct	4	—		179		185		283.3	3.4
Spco	2007	cT	5	—		161	2	171	0	296	12.5
Spco	2007	Ct	4	—		166.5	2.9	180	5.5	281.5	3.5
Spco	2007	CT	5	—		159.0*	0	171	0	286.4	3.4
Spco	2008	ct	4	127		—		—		299.3	6.5
Spco	2008	cT	5	113.6		205		234		308	3.7
Spco	2008	Ct	5	126.4		177		184		290.6	4.2
Spco	2008	CT	5	112.8		170		177		306.8	3.2
Spco	2009	ct	1	84		—		—		—	
Spco	2009	cT	3	78.7		161		—		286	7
Spco	2009	Ct	0	—		—		—		—	
Spco	2009	CT	1	76.0*		—		—		—	
Spco	2010	ct	5	—		190	11	211.5	2.5	290.8	1.2
Spco	2010	cT	5	71.5		178.8	4	210.3	1.3	288.4	1.5
Spco	2010	Ct	5	—		—		—		285.2	4.3
Spco	2010	CT	5	66.0*		176.5	2.5	211.5	2.5	282.8	3.5
Spco	2011	ct	5	115		189		196		284.4	3.4
Spco	2011	cT	5	87.7		176.4	1.9	186.6	0.6	287.2	4.8
Spco	2011	Ct	5	108		—		—		274.6	1.4
Spco	2011	CT	5	99		168	7	186	0	276.4	3.4
Treatment designation are as follows: ct are the ambient CO_2_, ambient temperature treatments (i.e., control); cT are the ambient CO_2_, warmed treatments; Ct are the CO_2_ enriched, ambient temperature treatments; and CT are the CO_2_ enriched, warmed treatments. Marked (*) cells designate those species that initiated leaf emergence and flower production earliest, and seed maturation and senescence latest in the season within a treatment within a year. Standard errors (*SE*) are included in parenthesis where applicable. Where *SE* is blank, there was no variation in induction dates for that particular species or the sample size was not more than one individual per treatment per year. Number of plots (n) where species did occur per treatment is also included (maximum possible n is 5).											
